# Effects of alkaline mineral complex supplementation on production performance, serum variables, and liver transcriptome in calves

**DOI:** 10.3389/fvets.2023.1282055

**Published:** 2023-12-06

**Authors:** Cheng Guo, Xiaowei Wang, Dongwen Dai, Fanlin Kong, Shuo Wang, Xiaoge Sun, Shengli Li, Xiaofeng Xu, Lili Zhang

**Affiliations:** ^1^College of Animal Science and Technology, Ningxia University, Yinchuan, China; ^2^State Key Laboratory of Animal Nutrition, Beijing Engineering Technology Research Center of Raw Milk Quality and Safety Control, College of Animal Science and Technology, China Agricultural University, Beijing, China

**Keywords:** calf, stress, anti-inflammatory activity, antioxidant capacity, liver transcriptome

## Abstract

Calf diarrhea causes huge economic losses to livestock due to its high incidence and mortality rates. Alkaline mineral complex water is an alkaline solution containing silicon, sodium, potassium, zinc, and germanium, and has biological benefits and therapeutic effects. This study aimed to evaluate the impact of alkaline mineral complex water supplementation on the health of calves and to investigate the effect of Alkaline mineral complex water supplementation on neonatal calf serum variables and the liver transcriptome. Sixty Holstein calves (age 1.88 ± 0.85 days, weight 36.63 ± 3.34 kg) were selected and randomly divided into two groups: the T group (treatment group with alkaline mineral complex water supplemented during the experiment) and C group (control group without alkaline mineral complex water supplementation). Alkaline mineral complex water supplementation significantly increased the body weight for calves aged 60 d and average daily gain during the experimental period (1–60 d). In addition, Alkaline mineral complex water supplementation could significantly decrease the diarrhea rate for calves aged 16–30 d, enhance the T-AOC, IgG, IGF-1, and IGFBP-2 in concentrations. The results of KEGG enrichment analysis in transcriptomics indicate that Alkaline mineral complex water supplementation inhibited the target IL-1B gene of the NF-kappa B signaling pathway of liver. Alkaline mineral complex water supplementation decreased calf diarrhea and improved partial immune function, anti-inflammatory activity, antioxidant capacity, and health of calves. Alkaline mineral complex is a candidate to replace medicated feed additives. Alkaline mineral complex waterAlkaline mineral complex waterAlkaline mineral complex waterAlkaline mineral complex waterAlkaline mineral complex waterAlkaline mineral complex waterAlkaline mineral complex water.

## Introduction

In contemporary farm production, it is well-established that farm animals undergo a series of stressors post-birth, significantly impacting their overall welfare. These stressors encompass a range of factors, including separation from maternal animals (1), the stress of transportation (2), challenges posed by the farm environment (3), the stress of weaning (4), antibiotic usage (5), heat stress (6) and adjustments in their dietary regimen (6). Newborn animals, in particular, exhibit heightened vulnerability to these stressors and require a higher level of care and attention to ensure their well-being (7, 8). Adequate nutrition of calves is a fundamental requirement for efficient production in later life. Suboptimal nutrition before weaning could have detrimental long-term effects on metabolic health and could thereby decrease production efficiency (7).

Water is an essential component of the diet and plays an important role in animal nutrition (9). Natural alkaline mineral water usually contains sodium, potassium, zinc, metasilicic acid, and other rare minerals (10). These alkaline minerals are rich in inorganic components in biota and play key roles in physiological metabolism and catalysis (11). Alkaline mineral water or mineral supplementation can improve the quality of life of patients with diarrhea-predominant irritable bowel syndrome and improve the gut health of young animals (12, 13). Calf diarrhea (CD) is a common stress disease associated with livestock production (14). Calf diarrhea causes huge economic losses to livestock due to its high incidence and mortality rates (15, 16). Antibiotics are routinely used to treat CD (17); however, excessive use of antibiotics can lead to various side effects, the most significant of which is infection by multidrug-resistant bacteria, which is increasing world-wide (18). Therefore, it is critical to find a low-cost, convenient and without side effect method to improve CD and calf immunity.

Alkaline mineral complex (AMC) water has biological benefits and therapeutic effects, including improving the quality of life of patients with cancer, conferring anti-oxidative effects, promoting intestinal health, and treating intestinal inflammatory diseases and diarrhea (12, 19, 20). The Alkaline mineral complex water used in this study was an alkaline solution (pH 9.1) containing silicon, sodium, potassium, zinc, and germanium, and its functions were based on its mineral composition (21). These minerals are essential for various physiological functions [such as digestion and immune biosynthesis in animals (22, 23)]. The liver is a key metabolic and central immune organ (24). Research has shown that the liver responds to these minerals, affecting their immune function (25). Recent research has shown that Alkaline mineral complex water can improve piglet diarrhea. Study of Chen et al. (26) showed that Alkaline mineral complex water (as used in this study) can improve intestinal morphology and inflammatory reactions, promote intestinal health, and accelerate the growth performance of weaned piglets. In addition, Alkaline mineral complex waterAlkaline mineral complex water can improve the structure and function of intestinal microflora, maintain intestinal epithelial regeneration, and improve the ability of piglets to recover from diarrhea (21, 27). In this experiment, we expected Alkaline mineral complex water supplementation to improve CD, enhance partial immune function, and promote their growth, development, and health. As calves are born with an esophageal sulcus reflex, their digestive mode is similar to that of non-ruminants (28). At present, it is known that Alkaline mineral complex water could improve diarrhea and intestinal immune function. However, as the largest immune organ in the liver, it is unclear how the expression of liver function genes and participation in immunity can be enhanced by Alkaline mineral complex water supplementation. In addition, the value of AMC in ruminants has not yet been explored. There has been no research on AMC in ruminants, and the effect of Alkaline mineral complex water on the liver transcriptome has not yet been studied. Hence, our study aimed to evaluate the impact of Alkaline mineral complex water supplementation on the health of calves and to investigate the effect of Alkaline mineral complex water supplementation on calf serum variables and the liver transcriptome.

## Materials and methods

All experimental methods and humane endpoints for decreasing pain in animals were performed after approval by the Experimental Animal Welfare and Animal Experiment Ethics Review Committee of China Agricultural University (Approval No. AW01103202-1-32). Animal research was conducted in accordance with State Council Order No. 676 of the People’s Republic of China (Regulations on the Administration of Experimental Animals).

### Experimental animals and treatment headings

The experiment was conducted on a commercial dairy farm in Yinchuan, Ningxia, China. According to the principle of similar body weight (BW), and age (1–3 d), 60 neonatal Holstein calves aged 1.88 ± 0.85 d (mean ± SD) and weighing 36.63 ± 3.34 kg BW were selected. Briefly, according to a completely randomized design, 60 neonatal calves were randomly divided into two groups in the experimental cattle farm: the T group (treatment group with alkaline mineral complex water supplemented during the experiment) and C group (control group without alkaline mineral complex water supplementation), with 30 calves (including 10 male calves and 20 female calves) in each group. All experimental calves were housed in individual hutches (each hutch has an area of at least 2.5 × 1.6 m). Calves could have direct visual and tactile contact, and have hay bedding. Calves were fed at 06:00 and 16:00 daily on the farm, and the daily liquid feed rate during the experimental period was 4 L/calf at 1–7 d of age, 6 L/calf at 8–14 d, 8 L/calf at 15–25 d, and 10 L/calf at 26–50 d; this was reduced by 2 L/calf every 2 d between 51 and 60 days of age until weaning. Pelleted feed was added to calves at 31 days of age. The water and pellets were changed daily during feeding to ensure the pellets were clean and fresh. To ensure that there was no interference from other mineral elements in this experiment, pure drinking water was used. The experiment included 7 d of adaptation, followed by 53 d (until weaning) of formal experimentation. During the 16–30 d experimental period, two calves in the C group died because they were unable to recover from diarrheal symptoms.

### Experimental feed

Experimental diet according to Nutrient Requirements of Dairy Cattle (NRC) (29). The ingredients and nutrient compositions of the experimental diets and liquid feed are listed in Supplementary Tables S1–S3. AMC is a liquid colloidal substance that is soluble in water, and it contains Na_2_SiO_3_, ZnO, Ge-132. Alkaline mineral complex water was supplemented using a continuous syringe during feeding in the afternoon during the experimental period (Supplementary Figure S1). The calves were fed twice at 06:00 and 16:00 and had free access to water. The liquid feed for 1–7 d of age of calves was fed in bottles with rubber nipples, and the calves were guided to use buckets for liquid feed. Calves after 8 days of age consume liquid feed in buckets. According to Chen et al. (26), based on the nutritional needs of calves, Alkaline mineral complex water supplementation should be 5 mL per cow per day. All the raw materials for AMC were provided by Nail Biotechnology Co., Ltd. (Beijing, China). The compositions of the AMC are listed in Table 1. Both pellet feed (Borui Technology Co., Ltd., Changchun, China) and feeding milk (Nutrifeed Co., Ltd., Veghel, Netherlands) were commercial feed. The mixing ratio of feeding milk was: (milk replacer 1: water 7): normal milk =1:1. All feed implemented Current National Standards and Industry Standards for Feed Industry of China. The feed provided in this study was carefully monitored to ensure that aflatoxin levels were well below the established safety limits for animal feed.

**Table 1:** The composition of alkaline mineral complex (AMC) concentrate.

Ingredient	Content	Chemical formula
Sodium metasilicate pentahydrate	200 g/L	5H_2_O·Na_2_SiO_3_
Potassium bicarbonate	100 g/L	KHCO_3_
Zinc oxide	10 mg/L	ZnO
Bis-(carboxyethylgermanium) sesquioxide	1 mg/L	Ge-132

### Growth performance measurements and diarrhea rate statistics

BW was measured at birth and every month thereafter. Body weight was measured by weighing the calves 2 h before the morning feeding (when the calves’ stomachs were empty). The weight of the pellet feed was measured before feeding (06:00) every day, and the pellet feed intake of the previous day for calves was calculated. The severity of diarrhea in the experimental calves was graded according to the consistency of feces. The fecal scores were as follows:

1 = normal (retains shape), 2 = soft (flows across a surface), 3 = muddy (liquid), and 4 = severe diarrhea (very watery) (14).

Diarrhea rate (%) = (total number of calves with diarrhea × days of diarrhea)/(total number of experimental calves × experimental period days) × 100 (30).

One independently trained observer collected the fecal score data. Calves were defined as having diarrhea with scores of 3 or 4.

### Blood samples collection and analysis

Ten calves from each experimental group were randomly selected for blood analysis. Blood samples were collected as described by Liu et al. (30). On days 1, 15, 30, 45, and 60 of the experimental period, blood was collected through jugular puncture using a vacuum blood collection vessel without an anticoagulant 2 h after morning feeding. Blood samples were centrifuged at 3,000 × g for 15 min at 4°C to collect serum and stored in 1.5 mL microcentrifuge tubes at −80°C for later analysis.

According to the radioimmunoassay method, insulin (INS) and growth hormone (GH) were analyzed with a measuring instrument (BFM-96, Zhongcheng Electromechanical Technology Development Co., Ltd., Hefei, China). Total cholesterol (TC), blood urea nitrogen (BUN), alkaline phosphatase (ALP), total protein (TP), albumin (ALB), globulin (GLB), malondialdehyde (MDA), glutathione peroxidase (GSH-PX), superoxide dismutase (SOD) and total antioxidant capacity (T-AOC) were determined according to the manufacturer’s instructions, using commercial reagent kits (Nanjing Jiancheng Co., Ltd., Nanjing, China) and an automatic biochemical analyzer (CLS880, Zecen Biotechnology Co., Ltd., Jiangsu, China). The β-hydroxybutyric acid (BHBA), nonestesterified fatty acid (NEFA), immunoglobulin G (IgG), insulin-like growth factor-1 (IGF-1), insulin-like growth factor binding protein-2 (IGFBP-2), zonula occludens-1 (ZO-1), endothelin-1 (ET-1), diamine oxidase (DAO) and D-lactic acid (D-LA) were analyzed using ELISA kits (Abcam, Cambridge, U K) with a microplate reader (Thermo Multiskan Ascent, Thermo Fisher Scientific, Shanghai, China).

### Collection, RNA extraction, sequencing and differential expression gene and function analysis of liver samples

Ten calves were randomly selected for humane slaughter at 60 d of the experimental period (five calves in Group C and five calves in Group T). After slaughtering, liver samples were collected from the same areas of calves in the C and T groups. Liver sample collection was done according to the method of Zhao et al. (31). Briefly, liver samples were quickly sectioned, washed with precooled PBS (4°C), placed in 2 mL sterile cryopreservation tubes, quickly frozen in liquid nitrogen, and then stored at −80°C for further analysis.

According to the manufacturer’s instructions (Invitrogen, Carlsbad, CA, United States), total RNA was extracted from liver tissue using TRIzol^®^ Reagent and genomic DNA was removed using DNase I (TaKara, Kusatsu, Japan). RNA degradation and contamination were monitored by 1% agarose gel electrophoresis. RNA quality was determined using a 2,100 Biological Analyzer (Agilent Technologies, Santa Clara, CA, United States) and quantified using an ND-2000 (NanoDrop Technologies, Wilmington, DE, United States). Only high-quality RNA samples (OD260/280 = 1.8–2.2, OD260/230 ≥ 2.0, RIN ≥ 8.0, 28S:18S ≥ 1.0 > 1 μg) were used to construct the sequencing library.

RNA purification, reverse transcription, library construction, and sequencing were performed at Shanghai Majorbio Bio-pharm Biotechnology Co., Ltd. (Shanghai, China), according to the manufacturer’s instructions (Illumina, San Diego, CA, United States). The transcriptome library was prepared using the TruSeqTM RNA sample preparation Kit from Illumina (San Diego, CA, USA) using 1 μg of total RNA. Briefly, mRNA was isolated according to the poly(A) selection method using oligo(dT) beads and then fragmented using fragmentation buffer. Next, double-stranded cDNA was synthesized using a SuperScript double-stranded cDNA synthesis kit (Invitrogen) with random hexamer primers (Illumina). Then the synthesized cDNA was subjected to end-repair, phosphorylation and ‘A’ base addition, according to Illumina’s library construction protocol. Libraries were selected for cDNA target fragments of 300 bp on 2% low-range ultra-agarose, followed by PCR amplification using Phusion DNA polymerase (NEB) for 15 PCR cycles. After quantification using TBS380, the paired-end RNA-seq library was sequenced using an Illumina NovaSeq 6000 sequencer (2 × 150 bp read length).

### Data analysis

The data displayed in the tables and figures represent the mean ± standard error of the mean (SEM). Before conducting statistical analysis, the normality and homogeneity of data of average daily gain, feed intake and serum variables were tested based on the description of Marta et al. (32). The effects of Alkaline mineral complex water supplementation on serum variables in neonatal calves were assessed using SAS software (version 9.4; SAS Institute Inc., Cary, NC, United States). According to the following model, a block experimental design was used for the time, group, and interaction effects between treatment and group:

$Y=\mu +T_{i}+G_{j}+{\mathrm{TG}}_{\mathrm{ij}}+E_{\mathrm{ijl}}$

*Y* is the dependent variable, *μ* is the overall mean, *T_i_* is the time effect, *G_j_* is the treatment effect, TG_ij_ is the interaction effect between *T* and *G*, *E*_ijl_ is the random residual error. Statistical significance was set at *p* < 0.05. different. One-way analysis of variance using SAS 9.4, the statistical significance of the effect of Alkaline mineral complex water supplementation on se-rum variables. Differences were considered statistically significant at *p* < 0.05, and trends were declared at 0.05 ≤ *p* ≤ 0.10.

The source of the reference gene was *Bos taurus*, the version of the reference genome was ARS-UCD1.2, and the source of the reference genome was http://asia.ensembl.org/Bo. ctaurus/Info/Index. The Clean Reads of each sample were sequenced with the specified reference genome. Transcriptome analysis of liver samples was performed using the Majorbio Cloud Platform (www.majorbio.com) (33). Differential expression analysis was performed using DESeq2, and DEGs with log2(fold change) ≥ 1 and P-adjusted ≤0.05 (DESeq2) were considered to be significant DEGs. Kyoto En-cyclopedia of Genes and Genomes (KEGG) pathway enrichment analysis was per-formed using R software (version 3.3.1). The KEGG pathway function was considered significantly enriched when the *p*-value was <0.05. All other statistical analyzes were performed using GraphPad Prism version 9 (GraphPad Software).

## Results

### Diarrhea rate of calves

**Figure 1:**
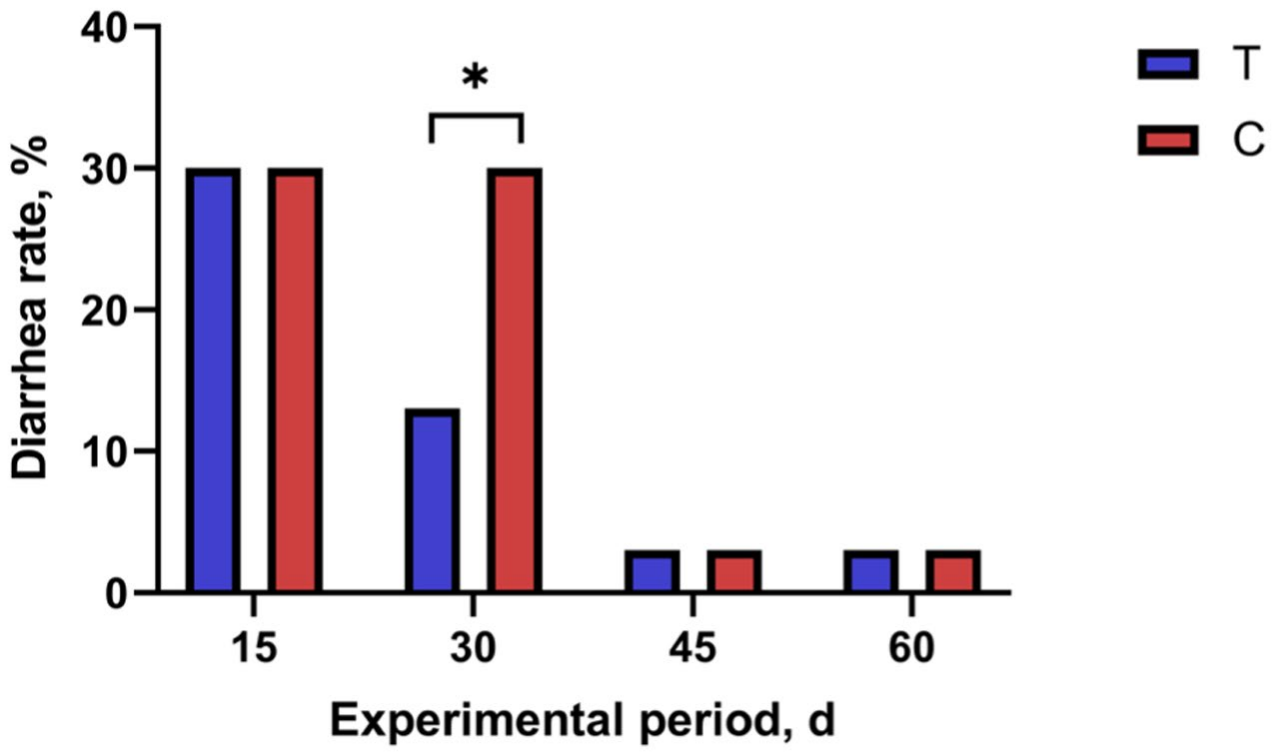
Effects of alkaline mineral complex water supplementation on diarrhea rate of calves. C is the control group; T is the treatment group. *p < 0.05. *n* = 30 per experimental group.

The effect of alkaline mineral complex water supplementation on the diarrhea rate is shown in Figure 1. Alkaline mineral complex water supplementation significantly decreasedthe diarrhea rate for calves aged 16–30 d (*p* < 0.05).

### Average daily gain and feed intake of calves

**Figure 2:**
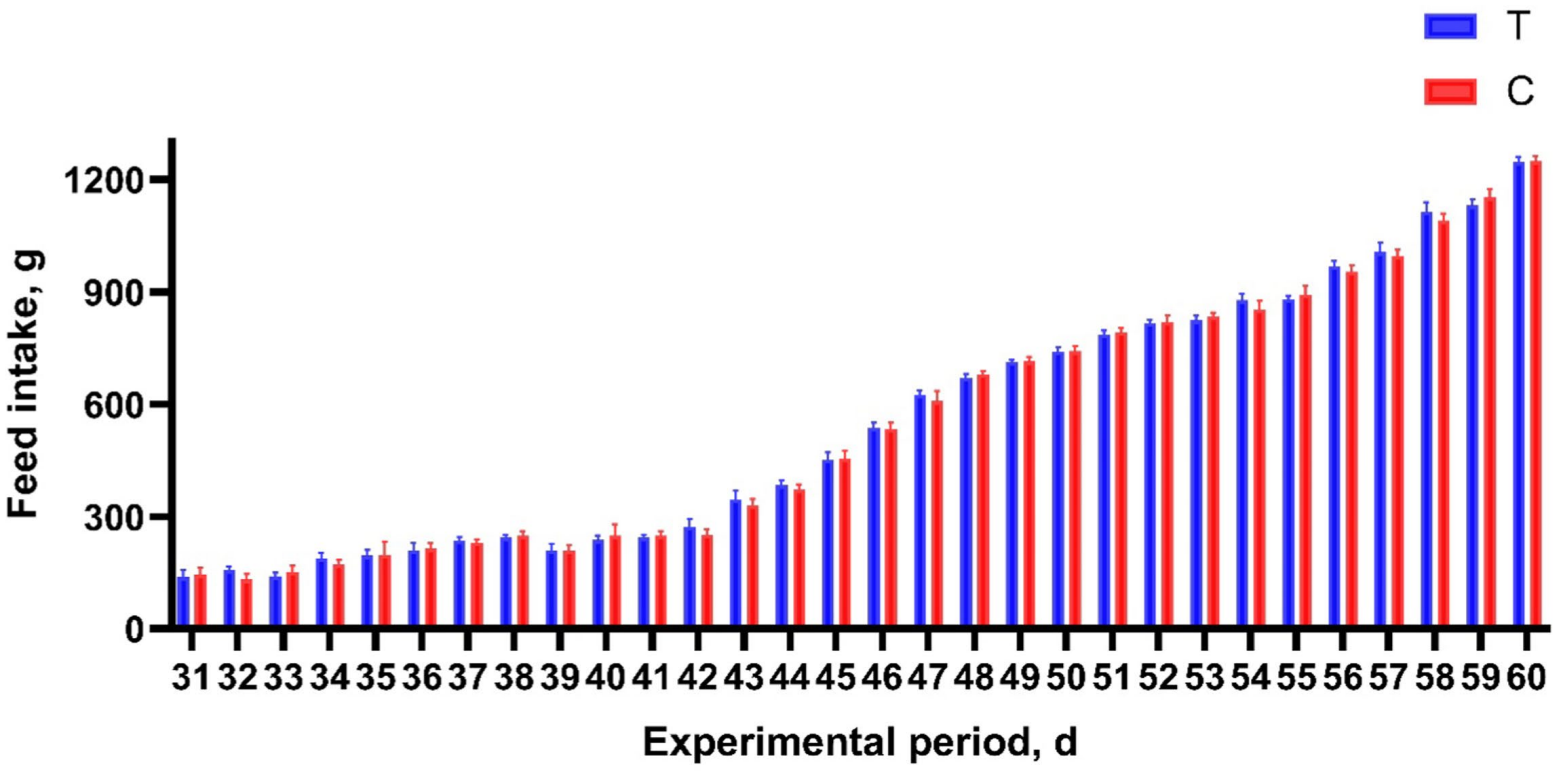
Effects of alkaline mineral complex water supplementation on pellets feed intake of calves. C is the control group; T is the treatment group. *n* = 30 per experimental group.

Alkaline mineral complex water supplementation for pellet feed intake is presented in Table 2 and Figure 2. Alkaline mineral complex water supplementation for BW and average daily gain (ADG) are presented in Table 3. AMC supplementation significantly increased the BW for calves aged 60 d (*p* < 0.05) and ADG during the experimental period (1–60 d) (*p* < 0.05). In addition, due to the natural characteristics of calves, they prefer milk more. All liquid feed calves fed daily can be consumed.

**Table 2:** Effects of alkaline mineral complex water supplementation on pellets feed intake in calves.

Items	Experimental period	C^a^	T^b^	SEM	*p*-value
Pellets feed intake, g	30–40 d	190.23	203.23	29.96	0.66
	40–50 d	493.60	498.60	28.42	0.86
	50–60 d	963.00	964.80	20.09	0.95
Total pellets feed intake, g	30–60 d	548.94	555.55	16.71	0.69

a C, Control group.

b T, Treatment group.

Data are presented as the means ± SEM (*n* = 30 per experimental group).

**Table 3:** Effects of alkaline mineral complex water supplementation on body weight and average daily gain in calves.

Items	Experimental period	C^a^	T^b^	SEM	*p*-value
Body weight, kg	1 d	36.00	36.65	2.00	0.75
	30 d	65.13	67.38	1.49	0.40
	60 d	103.40	107.50	1.92	0.03
Average daily gain, kg/d	1–30 d	0.81	1.00	0.11	0.08
	30–60 d	1.22	1.23	0.05	0.95
	1–60 d	1.11	1.22	0.04	0.01

a C, Control group.

b T, Treatment group.

Data are presented as the means ± SEM (*n* = 30 per experimental group).

### Serum variables of calves

**Figure 3:**
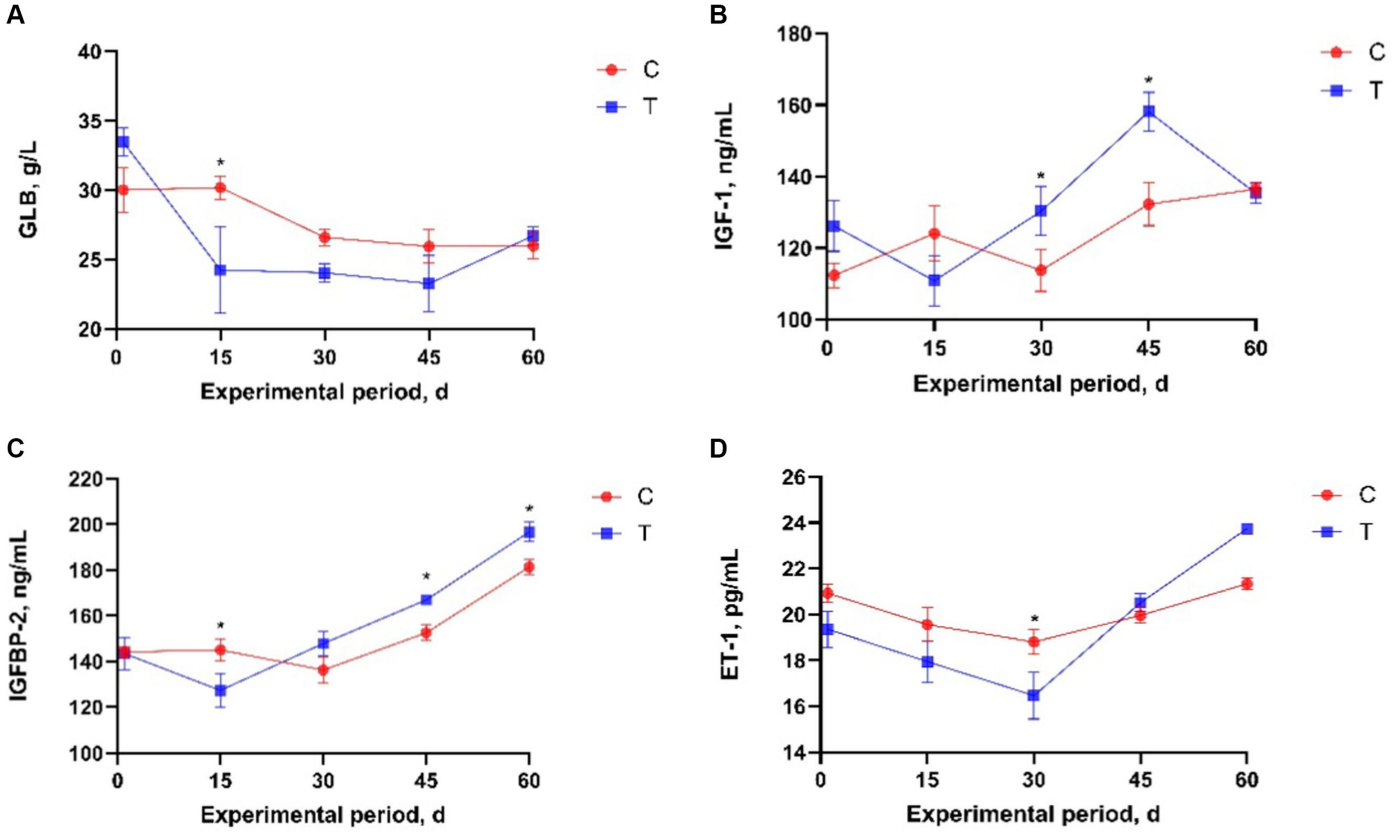
Effects of alkaline mineral complex water supplementation on serum variables in calves. **(A)** GLB, **(B)** IGF-1, **(C)** IGFBP-2, **(D)** ET-1. C is the control group; T is the treatment group. Data are presented as the means ± SEM (*n* = 10 per experimental group). *p < 0.05.

The effects of alkaline mineral complex water supplementation on serum variables are presented in Table 4. Compared to calves in the T and C groups, alkaline mineral complex water supplementation significantly reduced BUN levels during the experimental period (*p* < 0.05) and significantly increased T-AOC and IgG levels (*p* < 0.05). A significant interaction was observed between the experimental groups and AMC supplementation time for GLB, IGF-1, IGFBP-2, and ET-1 (*p* < 0.05). Further analysis of serum variable data revealed that alkaline mineral complex water supplementation significantly increased IGF-1 at 30 and 45 days of the experimental period and IGFBP-2 at 45 d of experimental period (*p* < 0.05) (Figure 3), while significantly decreasing GLB and IGFBP-2 at 15 d and ET-1 at 30 d of experimental period (*p* < 0.05) (Figure 3).

**Table 4:** Effects of alkaline mineral complex water supplementation on serum variables in calves.

Item	Group		SEM	*p*-value	
	T^a^	C^b^		Group	Group*Time
INS, mIU/mL	28.09	27.79	1.34	0.82	0.37
GH, ng/mL	1.34	1.24	0.07	0.16	0.29
GLU, mmol/L	7.46	7.43	0.15	0.84	0.07
TC, mmol/L	2.87	3.05	0.12	0.13	0.86
BUN, mmol/L	2.94	3.30	0.14	0.01	0.35
TP, g/L	55.20	55.39	0.90	0.84	0.09
ALB, g/L	27.69	27.89	0.27	0.46	0.63
GLB, g/L	26.36	27.75	0.86	0.11	0.02
MDA, nmol/mL	1.52	1.50	0.03	0.54	0.83
GSH-PX, umol/L	7.06	6.87	0.28	0.49	0.48
SOD, U/mL	46.48	47.33	0.90	0.35	0.64
T-AOC, U/mL	8.30	7.87	0.18	0.02	0.15
NEFA, umol/L	44.92	44.71	0.76	0.77	0.19
BHBA, mmol/L	0.36	0.35	0.01	0.63	0.07
IgG, mg/mL	21.09	20.09	0.31	<0.01	0.13
IGF-1, ng/mL	132.16	123.81	4.56	0.07	0.02
IGFBP-2, ng/mL	156.45	151.87	3.14	0.15	0.01
ZO-1, ng/mL	27.79	27.80	0.01	0.99	0.67
ET-1, pg./mL	19.60	20.11	0.14	0.27	0.03
DAO, U/mL	3.87	3.95	0.08	0.67	0.17
D-LA, umol/L	6.31	6.35	0.05	0.69	0.05

a C, Control group.

b T, Treatment group.

Data are presented as the means ± SEM (*n* = 10 per experimental group).

### Differentially expressed genes in the liver of C and T groups

**Figure 4:**
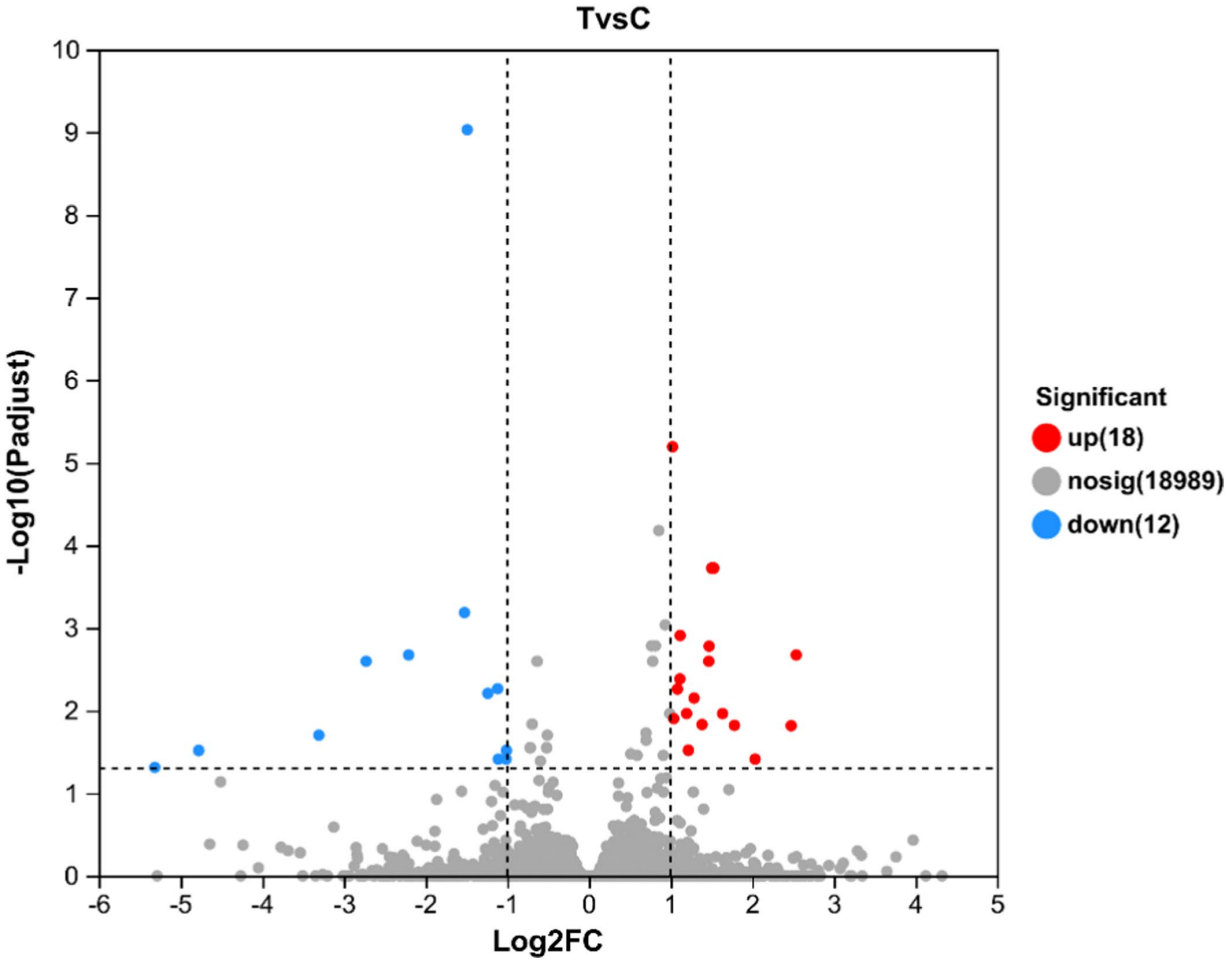
Volcano map of differentially expressed genes in the liver of calves at 60 d of the experimental period. The horizontal coordinate is the multiple of gene/transcript expression difference between the two samples, namely the value obtained by dividing the amount expressed by treated samples by the expression amount of control samples; the vertical coordinate is the statistical test value of gene expression difference, namely the *p* value. The larger the −log10(*p*-value) is, the more significant the difference in ex-pression is. Both the horizontal and vertical values are logized. Each dot in the figure represents a specific gene, with red, blue, and gray dots representing significantly up-regulated, significantly downregulated, and non-significant genes, respectively. The points on the left and right are genes with downregulated and upregulated expression, respectively. The closer to the two sides and the point on the top, the more significant the expression difference.

A total of 19,019 genes were detected and tested for differential expression in the liver samples, and 30 were differentially expressed between the T and C groups (*p* < 0.05) (Figure 4). Table 3 lists the significant DEGs in the liver samples of the calves; some unnamed genes are not listed. Respectively, DDIT4, TAT, NOCT, FOSB, SPRY3, LU-RAP1L, ARHGEF26, DUSP1, FHIT, et al. and GSTT4, IL1B, DUSP6, MID1IP1, OSGIN1, COBL, TFAP4, et al. transcripts were up and downregulated in the T group compared to the C group (Table 5).

**Table 5:** Significantly differentially expressed genes in the liver samples of calves at 60 d of the experimental period.

Gene id	Gene name	Log2FC (T/C)	*p* value	P-adjust	Regulate
ENSBTAG00000000163	DDIT4	1.50	6.00E-08	1.89E-04	Up
ENSBTAG00000000170	GSTT4	−3.31	5.59E-05	0.02	Down
ENSBTAG00000001321	IL1B	−1.12	9.87E-06	0.01	Down
ENSBTAG00000002214	TAT	1.02	8.03E-10	6.42E-06	Up
ENSBTAG00000004587	DUSP6	−1.49	5.87E-14	9.38E-10	Down
ENSBTAG00000005998	NOCT	2.04	1.53E-04	0.04	Up
ENSBTAG00000008182	FOSB	2.47	3.92E-05	0.02	Up
ENSBTAG00000010245	SPRY3	1.20	2.24E-05	0.01	Up
ENSBTAG00000010431	LURAP1L	1.47	1.67E-06	1.66E-03	Up
ENSBTAG00000011463	MID1IP1	−1.52	2.85E-07	0.65E-03	Down
ENSBTAG00000012508	OSGIN1	−1.01	1.02E-04	0.03	Down
ENSBTAG00000013439	ARHGEF26	1.04	2.90E-05	0.01	Up
ENSBTAG00000013863	DUSP1	1.53	7.09E-08	1.89E-04	Up
ENSBTAG00000014418	FHIT	2.54	2.65E-06	2.12E-03	Up
ENSBTAG00000017162	STK39	1.64	2.38E-05	0.01	Up
ENSBTAG00000021672	RGS1	1.47	3.70E-06	2.53E-03	Up
ENSBTAG00000023806	COBL	−1.24	1.20E-05	6.20E-03	Down
ENSBTAG00000033174	TFAP4	−1.01	1.54E-04	0.04	Down
ENSBTAG00000039819	RPH3AL	1.39	3.61E-05	0.01	Up
ENSBTAG00000047103	IDNK	1.29	1.41E-05	0.01	Up
ENSBTAG00000052499	RNF39	−2.21	2.64E-06	2.12E-03	Down
ENSBTAG00000054218	IGFBP5	1.22	9.81E-05	0.03	Up

### KEGG enrichment analysis

**Figure 5:**
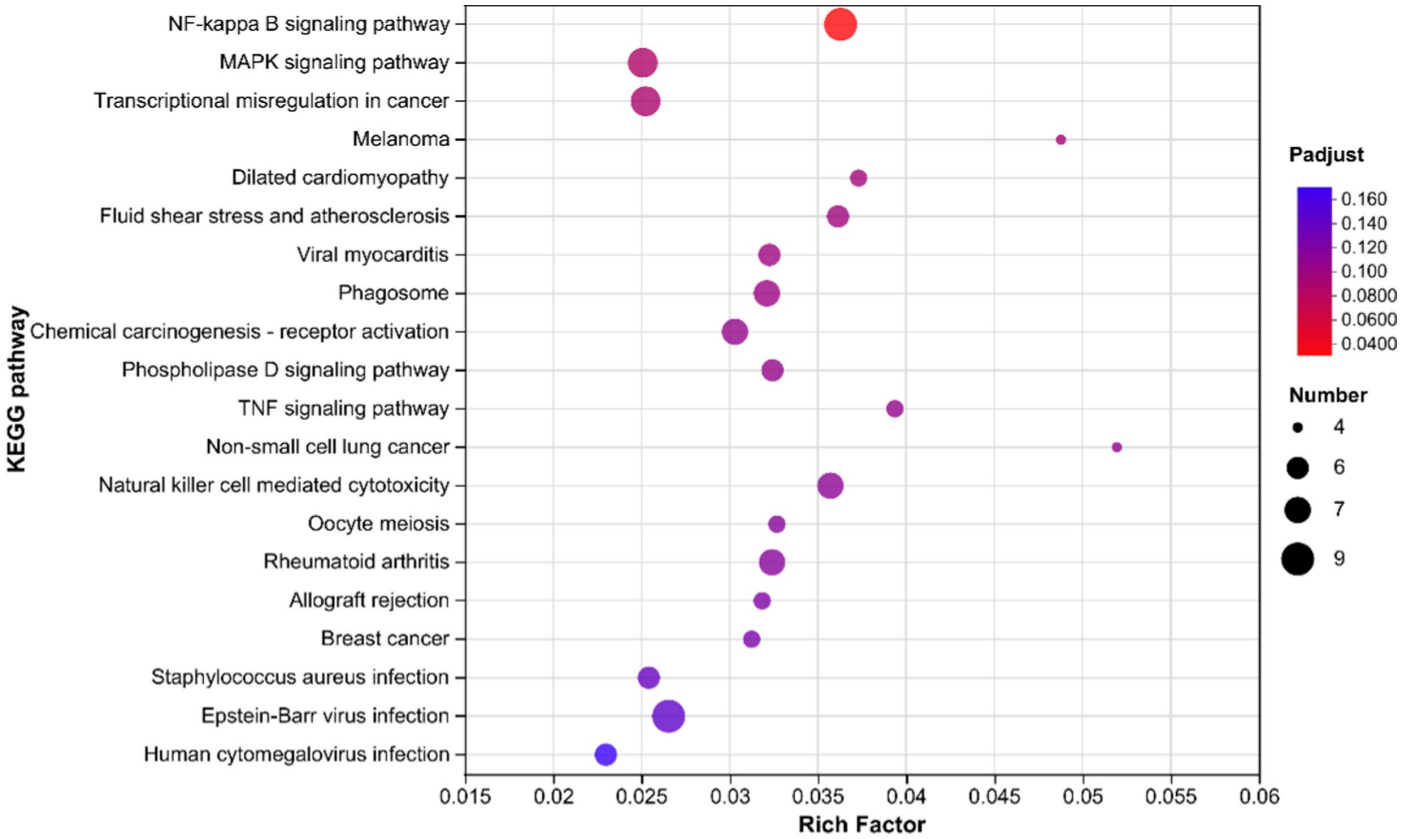
Kyoto Encyclopedia of Genes and Genomes enrichment analysis in the liver samples of 60 days of the experimental period of calves. The vertical axis represents the pathway names, and the horizontal axis represents the ratio of the enrichment factor (sample number of genes/transcripts enriched in this pathway to the background number of annotated genes/transcripts). The larger the enrichment factor, the greater the degree of enrichment. The size of dots indicates the number of genes in this pathway, and the colors of the dots correspond to different P-adjusted ranges. The enrichment results of top 20 were displayed when P-adjusted <0.05.

The KEGG enrichment analysis results for Alkaline mineral complex water supplementation are presented in Figure 5. The significant enrichment pathway was the NF-kappa B signaling pathway (P-adjusted <0.05). The MAPK signaling pathway and transcriptional mis-regulation in cancer tended to be significantly enriched, although these changes were not statistically significant (0.05 ≤ *p* ≤ 0.10).

## Discussion

Alkaline mineral complex water is rich in multiple trace elements. Although the role of AMC in non-ruminant is generally considered positive, its effectiveness in cattle farming has not yet been confirmed. In this study, we evaluated the impact of alkaline mineral complex water supplementation on the health of calves and investigated the effect of alkaline mineral complex water supplementation on production performance, serum variables, and the liver transcriptome. Our results indicated that alkaline mineral complex water supplementation could improve the health and promote the growth of calves.

The CD rate is related to age; calves are at a high risk for CD and death in early life, and CD in the first 3 weeks of life is a major cause of death (34, 35). With the growth and development of calves, their immunity improves and their CD rate decreases (36, 37). Alkaline mineral complex water contains silicates, which have anti-inflammatory properties and can effectively treat diarrhea (38). In this study, alkaline mineral complex water supplementation decreased the CD rate at 16–30 d of the experimental period. Alkaline mineral complex water supplementation had no significant effect in other experimental periods, possibly because Alkaline mineral complex water the immunity of calves improved with their growth and development after 30 d.

Feeding and management of calves at a young age are crucial for the growth, health, and future production performance of dairy cows. The BW of calves reflects their growth and development and is related to the incidence of diseases, reproductive performance, and milk yield (39). CD could have an impact on the BW of calves, which is negatively correlated with BW even after recovery from CD, and the ADG of calves could decrease (40). In this study, Alkaline mineral complex water supplementation increased BW and ADG. Feed palatability is a critical factor influencing feed intake and, subsequently, animal performance (41). In this experiment, calves could quickly consume all liquid feed every day due to their preference for milk. And there was no difference in the intake of pellet feed for calves. Therefore, AMC supplementation did not interfere with the normal feeding of calves in this experiment. In particular, Alkaline mineral complex water supplementation significantly increased the BW of calves aged 60 d and significantly increased the ADG during the experimental period (1–60 d). In this experiment, the decrease in CD and the increase in feed intake may be the reasons for the increase in calf BW and ADG. Increases in BW and ADG are of great significance for future calf production.

BUN is an important indicator of the balance between amino acid and protein metabolism in animals. Coma et al. (42) showed that animals with lower BUN concentrations had normal amino acid metabolism and higher protein synthesis rates. In this study, AMC supplementation significantly reduced BUN levels during the experimental period, which may indicate that AMC supplementation can improve the utilization rate of dietary protein in calves. This may be one of the reasons for the increase in BW and ADG during the experimental period.

Alkaline mineral complex water is an excellent source of silicon and zinc; these elements are related to the immune system and have anti-inflammatory and antioxidant effects (23, 43–45). Excessive serum globulin levels are associated with inflammation (46). Endothelial damage may increase ET-1 production (47). Alkaline mineral complex water supplementation significantly reduced GLB and ET-1, especially on days 15 and 30 of the experiment. Calves were most vulnerable and had the highest rates of CD in the period within 30 d after their birth. IgG can indicate the strength and weakness of the immune system, and the survival and well-being of calves are strongly dependent on IgG (48). Total antioxidant capacity represents the total antioxidant levels of various antioxidant substances and enzymes (49). In this study, AMC supplementation significantly increased T-AOC and IgG levels. Changes in these serum variables could imply that Alkaline mineral complex water has immunomodulator, anti-inflammatory, and antioxidant effects on calves and that alkaline mineral complex water supplementation improved their health. In addition, AMC supplementation promoted calf growth. AMC supplementation significantly increased IGF-1 and IGFBP-2 levels, particularly in the later period of the experiment. IGF-1 and IGFBP-2 are growth-promoting factors that costimulate osteoblast differentiation (50, 51); increases in BW and ADG confirmed this result. IGF signal transduction is regulated by conserved members of the IGFBP family. IGFBP-5 is a multifunctional protein that conditionally regulates IGF signaling as a molecular switch (52). In the present study, the increase in IGF-1 and IGFBP-2 levels in the treatment group may have been due to the significant upregulation of IGFBP-5. However, the specific regulatory mechanism of IGFBP-5 gene is still unclear and warrant further investigation.

The liver is the largest organ in the body and plays an important role in the immune system (53). NF-kappa B signaling pathway is a highly conserved evolutionary pathway that plays important functions in regulating immune and inflammatory responses (54). In the liver, NF-kappa B signaling pathway is an important transcriptional regulator of inflammatory response and plays an essential role in regulating liver inflammatory signaling pathways (55). In this study, alkaline mineral complex water supplementation significantly regulated the NF-kappa B signaling pathway in the liver. Chen et al. found that Alkaline mineral complex water could target the inhibition of NF-kappa B signaling pathway through microbial-intestinal interaction (27). IL-1B, one of the main cytokines involved in the pathogenesis of many inflammation-related diseases, and it can regulate the NF-kappa B signaling pathway (56, 57). In this study, alkaline mineral complex water supplementation significantly downregulated the IL-1B gene Research showed that trace elements that are abundant in alkaline mineral complex water could inhibit the IL-1B gene (58). This study showed that alkaline mineral complex water supplementation could inhibit the target genes of the NF-kappa B signaling pathway, affected the immune function of the liver, and improved the health of calves.

Calves, especially female calves, play a crucial role in the future of any herd, making them an important component of dairy farms. Raising calves requires a significant investment of resources and time to achieve profitability (59). Therefore, raising calves requires consideration of cost. Some limitations need to be acknowledged. Although alkaline mineral complex can be proven beneficial for calves, it is necessary to continuously supplement alkaline mineral complex before weaning. If large-scale fed can reduce the number of days for alkaline mineral complex supplementation, it can save costs. The cost of commercial alkaline mineral complex supplementation in calves does not exceed 0.04 dollars per calf per d, indicating that alkaline mineral complex is more economical compared to regular antibiotics. In the future, emphasis should be placed on determining the number of days calves are supplemented with alkaline mineral complex to save more costs.

## Conclusion

Under the experimental conditions applied, Alkaline mineral complex water supplementation promoted the growth and health of the calves, possibly by enhancing T-AOC, IgG, IGF-1, and IGFBP-2, thereby improving growth-promoting factors, antioxidant status, and partial immune function in calves. These effects decrease CD and promote the growth of calves. It is worth noting that Alkaline mineral complex water supplementation could inhibit the target IL-1B gene of the NF-kappa B signaling pathway, affect the immune and anti-inflammatory functions of the liver, and improve the health of calves. Overall, alkaline mineral complex water supplementation decreased calf diarrhea and improved, partial immune function, anti-inflammatory activity, antioxidant capacity, and health of calves; thus, it is a candidate to replace medicated feed additives.

## Data availability statement

The datasets presented in this study can be found in online repositories. The names of the repository/repositories and accession number(s) can be found at: https://www.ncbi.nlm.nih.gov/bioproject; PRJNA1008432.

## Ethics statement

The animal study was approved by Experimental Animal Welfare and Animal Experiment Ethics Committee of China Agricultural University. The study was conducted in accordance with the local legislation and institutional requirements.

## Author contributions

CG: Conceptualization, Data curation, Investigation, Methodology, Writing – original draft. XW: Investigation, Methodology, Validation, Writing – review & editing. DD: Investigation, Resources, Writing – review & editing. FK: Writing – review & editing. SW: Writing – review & editing. XS: Data curation, Software, Writing – review & editing. SL: Funding acquisition, Project administration, Supervision, Writing – review & editing. XX: Methodology, Project administration, Resources, Writing – review & editing. LZ: Methodology, Resources, Writing – review & editing.

## References

[1] Webb LE Marcato F Bokkers E Verwer CM Wolthuis-Fillerup M Hoorweg FA. Impact of early dam contact on veal calf welfare. Sci Rep. (2022) 12:22144. doi: 10.1038/s41598-022-25804-z, PMID: 36550162 PMC9780250

[2] Sardi L Gastaldo A Borciani M Bertolini A Musi V Garavaldi A. Pre-Slaughter sources of fresh meat quality variation: the case of heavy pigs intended for protected designation of origin products. Animals (Basel). (2020) 10:2386. doi: 10.3390/ani10122386, PMID: 33327382 PMC7764830

[3] Roadknight N Mansell P Jongman E Courtman N Fisher A. Invited review: The welfare of young calves transported by road. J Dairy Sci. (2021) 104:6343–57. doi: 10.3168/jds.2020-19346, PMID: 33714583

[4] Vickery HM Neal RA Stergiadis S Meagher RK. Gradually weaning goat kids may improve weight gains while reducing weaning stress and increasing creep feed intakes. Front Vet Sci. (2023) 10:1200849. doi: 10.3389/fvets.2023.1200849, PMID: 37332741 PMC10270287

[5] Masebo NT Marliani G Cavallini D Accorsi PA Di Pietro M Beltrame A. Health and welfare assessment of beef cattle during the adaptation period in a specialized commercial fattening unit. Res Vet Sci. (2023) 158:50–5. doi: 10.1016/j.rvsc.2023.03.008, PMID: 36924635

[6] Giorgino A Raspa F Valle E Bergero D Cavallini D Gariglio M. Effect of dietary organic acids and botanicals on metabolic status and milk parameters in mid-late lactating goats. Animals (Basel). (2023) 13:797. doi: 10.3390/ani13050797, PMID: 36899655 PMC10000138

[7] Kenéz Á Koch C Korst M Kesser J Eder K Sauerwein H. Different milk feeding intensities during the first 4 weeks of rearing dairy calves: Part 3: Plasma metabolomics analysis reveals long-term metabolic imprinting in Holstein heifers. J Dairy Sci. (2018) 101:8446–60. doi: 10.3168/jds.2018-14559, PMID: 29935828

[8] Eckert E Brown HE Leslie KE DeVries TJ Steele MA. Weaning age affects growth, feed intake, gastrointestinal development, and behavior in Holstein calves fed an elevated plane of nutrition during the preweaning stage. J Dairy Sci. (2015) 98:6315–26. doi: 10.3168/jds.2014-9062, PMID: 26142851

[9] Miller JD Workman CL Panchang SV Sneegas G Adams EA Young SL. Water security and nutrition: current knowledge and research opportunities. Adv Nutr. (2021) 12:2525–39. doi: 10.1093/advances/nmab075, PMID: 34265039 PMC8634318

[10] Bertoni M Olivieri F Manghetti M Boccolini E Bellomini MG Blandizzi C. Effects of a bicarbonate-alkaline mineral water on gastric functions and functional dyspepsia: a preclinical and clinical study. Pharmacol Res. (2002) 46:525–31. doi: 10.1016/s1043661802002323, PMID: 12457626

[11] Nan H Zhao L Yang F Liu Y Xiao Z Cao X. Different alkaline minerals interacted with biomass carbon during pyrolysis: Which one improved biochar carbon sequestration? J Clean Prod. (2020) 255:120162. doi: 10.1016/j.jclepro.2020.120162

[12] Shin DW Yoon H Kim HS Choi YJ Shin CM Park YS. Effects of alkaline-reduced drinking water on irritable bowel syndrome with diarrhea: a randomized double-blind, placebo-controlled pilot study. Evid Based Complement Alternat Med. (2018) 2018:9147914. doi: 10.1155/2018/9147914, PMID: 29849734 PMC5925025

[13] Shannon MC Hill GM. Trace mineral supplementation for the intestinal health of young monogastric animals. Front Vet Sci. (2019) 6:73. doi: 10.3389/fvets.2019.00073, PMID: 30918894 PMC6424858

[14] Islam J Tanimizu M Shimizu Y Goto Y Ohtani N Sugiyama K. Development of a rational framework for the therapeutic efficacy of fecal microbiota transplantation for calf diarrhea treatment. Microbiome. (2022) 10:31. doi: 10.1186/s40168-021-01217-4 35184756 PMC8858662

[15] Huang MZ Cui DA Wu XH Hui W Yan ZT Ding XZ. Serum metabolomics revealed the differential metabolic pathway in calves with severe clinical diarrhea symptoms. Animals (Basel). (2020) 10:10. doi: 10.3390/ani10050769, PMID: 32354125 PMC7278412

[16] Wu YY Nie CX Xu C Luo RQ Chen HL Niu JL. Effects of dietary supplementation with multispecies probiotics on intestinal epithelial development and growth performance of neonatal calves challenged with *Escherichia coli* K99. J Sci Food Agric. (2022) 102:4373–83. doi: 10.1002/jsfa.11791, PMID: 35066866 PMC9303730

[17] Eibl C Bexiga R Viora L Guyot H Felix J Wilms J. The antibiotic treatment of calf diarrhea in four european countries: a survey. Antibiotics (Basel). (2021) 10:910. doi: 10.3390/antibiotics10080910, PMID: 34438960 PMC8388724

[18] Uddin TM Chakraborty AJ Khusro A Zidan B Mitra S Emran TB. Antibiotic resistance in microbes: History, mechanisms, therapeutic strategies and future prospects. J Infect Public Health. (2021) 14:1750–66. doi: 10.1016/j.jiph.2021.10.020, PMID: 34756812

[19] Costantino M Conti V Corbi G Filippelli A. Hydropinotherapy with sulphurous mineral water as complementary treatment to improve glucose metabolism, oxidative status, and quality of life. Antioxidants (Basel). (2021) 10:1773. doi: 10.3390/antiox10111773, PMID: 34829645 PMC8614851

[20] Pavelic K Hadzija M Bedrica L Pavelic J Dikic I Katic M. Natural zeolite clinoptilolite: new adjuvant in anticancer therapy. J Mol Med (Berl). (2001) 78:708–20. doi: 10.1007/s001090000176 11434724

[21] Chen J Zhao BC Dai XY Xu YR Kang JX Li JL. Drinking alkaline mineral water confers diarrhea resistance in maternally separated piglets by maintaining intestinal epithelial regeneration via the brain-microbe-gut axis. J Adv Res. (2022) 52:29–43. doi: 10.1016/j.jare.2022.12.008, PMID: 36539076 PMC10555785

[22] Bao YM Choct M. Trace mineral nutrition for broiler chickens and prospects of application of organically complexed trace minerals: a review. Anim Prod Sci. (2009) 49:269. doi: 10.1071/EA08204

[23] Jurkic LM Cepanec I Pavelic SK Pavelic K. Biological and therapeutic effects of ortho-silicic acid and some ortho-silicic acid-releasing compounds: New perspectives for therapy. Nutr Metab (Lond). (2013) 10:2. doi: 10.1186/1743-7075-10-2, PMID: 23298332 PMC3546016

[24] Li J Yin LM Wang L Li JZ Huang PF Yang HS. Effects of vitamin B6 on growth, diarrhea rate, intestinal morphology, function, and inflammatory factors expression in a high-protein diet fed to weaned piglets. J Anim Sci. (2019) 97:4865–74. doi: 10.1093/jas/skz338, PMID: 31679024 PMC6915213

[25] Kozeniecki M Ludke R Kerner J Patterson B. Micronutrients in liver disease: roles, risk factors for deficiency, and recommendations for supplementation. Nutr Clin Pract. (2020) 35:50–62. doi: 10.1002/ncp.10451, PMID: 31840874

[26] Chen J Xu YR Kang JX Zhao BC Dai XY Qiu BH. Effects of alkaline mineral complex water supplementation on growth performance, inflammatory response, and intestinal barrier function in weaned piglets. J Anim Sci. (2022) 100:100. doi: 10.1093/jas/skac251, PMID: 35913841 PMC9584155

[27] Chen J Xu XW Kang JX Zhao BC Xu YR Li JL. Metasilicate-based alkaline mineral water confers diarrhea resistance in maternally separated piglets via the microbiota-gut interaction. Pharmacol Res. (2023) 187:106580. doi: 10.1016/j.phrs.2022.106580 36436708

[28] Hegland RB Lambert MR Jacobson NL Payne LC. Effect of dietary and managemental factors on reflex closure of the esophageal groove in the dairy calf 1. J Dairy Sci. (1957) 40:1107–13. doi: 10.3168/jds.S0022-0302(57)94602-7

[29] NRC. Nutrient requirements of dairy cattle. 7th rev. ed. Washington, DC: Proceedings of the National Academy of Sciences of the United States of America (2001). 381 p.

[30] Liu S Ma JY Zhou J Wu JD Li JH Alugongo GM. Tributyrin supplementation in pasteurized waste milk: Effects on growth performance, health, and blood parameters of dairy calves. J Dairy Sci. (2021) 104:12496–507. doi: 10.3168/jds.2021-20645, PMID: 34593232

[31] Zhao XW Zhu HL Qi YX Wu T Huang DW Ding HS. Quantitative comparative phosphoproteomic analysis of the effects of colostrum and milk feeding on liver tissue of neonatal calves. J Dairy Sci. (2021) 104:8265–75. doi: 10.3168/jds.2020-20097, PMID: 33865590

[32] Marta P Marco T Andrea F Daniela F Damiano C. Effect of does parity order on litter homogeneity parameters. Ital J Anim Sci. (2020) 19:1188–94. doi: 10.1080/1828051X.2020.1827990

[33] Ren Y Yu G Shi C Liu L Guo Q Han C. Majorbio Cloud: A one-stop, comprehensive bioinformatic platform for multiomics analyses. Imeta. (2022) 1:1. doi: 10.1002/imt2.12 PMC10989754 38868573

[34] Kim HS Whon TW Sung H Jeong YS Jung ES Shin NR. Longitudinal evaluation of fecal microbiota transplantation for ameliorating calf diarrhea and improving growth performance. Nat Commun. (2021) 12:161. doi: 10.1038/s41467-020-20389-5, PMID: 33420064 PMC7794225

[35] Pempek J Trearchis D Masterson M Habing G Proudfoot K. Veal calf health on the day of arrival at growers in Ohio. J Anim Sci. (2017) 95:3863–72. doi: 10.2527/jas2017.1642 28992033

[36] Kertz AF Hill TM Quigley JR Heinrichs AJ Linn JG Drackley JK. A 100-Year Review: Calf nutrition and management. J Dairy Sci. (2017) 100:10151–72. doi: 10.3168/jds.2017-13062, PMID: 29153160

[37] Lora I Gottardo F Contiero B Dall AB Bonfanti L Stefani A. Association between passive immunity and health status of dairy calves under 30 days of age. Prev Vet Med. (2018) 152:12–5. doi: 10.1016/j.prevetmed.2018.01.009, PMID: 29559100 PMC7114084

[38] González R Sánchez De Medina F Martínez-Augustin O Nieto A Gálvez J Risco S. Anti-inflammatory effect of diosmectite in hapten-induced colitis in the rat. Br J Pharmacol. (2004) 141:951–60. doi: 10.1038/sj.bjp.0705710, PMID: 14993105 PMC1574279

[39] Rashad A Amin AH Mahdy AE Aziz MA El-Barbary A El-Hedainy D. The impact of milk suckling protocol and schedule on body weight and some morphometric measurements of Holstein heifers. Trop Anim Health Prod. (2022) 54:187. doi: 10.1007/s11250-022-03182-y, PMID: 35546214 PMC9095540

[40] Schinwald M Creutzinger K Keunen A Winder CB Haley D Renaud DL. Predictors of diarrhea, mortality, and weight gain in male dairy calves. J Dairy Sci. (2022) 105:5296–309. doi: 10.3168/jds.2021-21667 35346468

[41] Vinassa M Cavallini D Galaverna D Baragli P Raspa F Nery J. Palatability assessment in horses in relation to lateralization and temperament. Appl Anim Behav Sci. (2020) 232:105110. doi: 10.1016/j.applanim.2020.105110

[42] Coma J Carrion D Zimmerman DR. Use of plasma urea nitrogen as a rapid response criterion to determine the lysine requirement of pigs. J Anim Sci. (1995) 73:472–81. doi: 10.2527/1995.732472x, PMID: 7601781

[43] Kim EJ Bu SY Sung MK Kang MH Choi MK. Analysis of antioxidant and anti-inflammatory activity of silicon in murine macrophages. Biol Trace Elem Res. (2013) 156:329–37. doi: 10.1007/s12011-013-9829-y, PMID: 24092518

[44] Powell SR. The antioxidant properties of zinc. J Nutr. (2000) 130:1447S–54S. doi: 10.1093/jn/130.5.1447S 10801958

[45] Prasad AS. Zinc: an antioxidant and anti-inflammatory agent: role of zinc in degenerative disorders of aging. J Trace Elem Med Biol. (2014) 28:364–71. doi: 10.1016/j.jtemb.2014.07.019, PMID: 25200490

[46] Mohri M Sharifi K Eidi S. Hematology and serum biochemistry of Holstein dairy calves: age related changes and comparison with blood composition in adults. Res Vet Sci. (2007) 83:30–9. doi: 10.1016/j.rvsc.2006.10.017 17188315

[47] Cozza EN Gomez-Sanchez CE Foecking MF Chiou S. Endothelin binding to cultured calf adrenal zona glomerulosa cells and stimulation of aldosterone secretion. J Clin Invest. (1989) 84:1032–5. doi: 10.1172/JCI114226, PMID: 2547837 PMC329753

[48] Drikic M Windeyer C Olsen S Fu Y Doepel L De Buck J. Determining the IgG concentrations in bovine colostrum and calf sera with a novel enzymatic assay. J Anim Sci Biotechnol. (2018) 9:69. doi: 10.1186/s40104-018-0287-4, PMID: 30214721 PMC6131873

[49] Wang S Hu F Diao Q Li S Tu Y Bi Y. Comparison of growth performance, immunity, antioxidant capacity, and liver transcriptome of calves between whole milk and plant protein-based milk replacer under the same energy and protein levels. Antioxidants (Basel). (2022) 11:270. doi: 10.3390/antiox11020270, PMID: 35204153 PMC8868243

[50] Xi G Rosen CJ Clemmons DR. IGF-I and IGFBP-2 Stimulate AMPK activation and autophagy, which are required for osteoblast differentiation. Endocrinology. (2016) 157:268–81. doi: 10.1210/en.2015-1690, PMID: 26556533 PMC4701891

[51] Morimoto-Kamata R Tsuji D Yui S. Cathepsin g-induced insulin-like growth factor (IGF) elevation in MCF-7 medium is caused by proteolysis of IGF binding protein (IGFBP)-2 but not of IGF-1. Biol Pharm Bull. (2020) 43:1678–86. doi: 10.1248/bpb.b20-00389 33132312

[52] Duan C Allard JB. Insulin-like growth factor binding protein-5 in physiology and disease. Front Endocrinol (Lausanne). (2020) 11:100. doi: 10.3389/fendo.2020.00100, PMID: 32194505 PMC7063065

[53] Bogdanos DP Gao B Gershwin ME. Liver immunology. Compr Physiol. (2013) 3:567–98. doi: 10.1002/cphy.c120011 23720323 PMC4201126

[54] Xiao C Ghosh S. NF-kappa B, an evolutionarily conserved mediator of immune and inflammatory responses. Adv Exp Med Biol. (2005) 560:41–5. doi: 10.1007/0-387-24180-9_5 15932018

[55] Luedde T Schwabe RF. NF-κB in the liver-linking injury, fibrosis and hepatocellular carcinoma. Nat Rev Gastroenterol Hepatol. (2011) 8:108–18. doi: 10.1038/nrgastro.2010.213, PMID: 21293511 PMC3295539

[56] Zhang H. Anti-IL-1beta therapies. Recent Pat DNA Gene Seq. (2011) 5:126–35. doi: 10.2174/187221511796392024 21762108

[57] Cheng CY Hsieh HL Sun CC Lin CC Luo SF Yang CM. IL-1 beta induces urokinase-plasminogen activator expression and cell migration through PKC alpha, JNK1/2, and NF-kappa B in A549 cells. J Cell Physiol. (2009) 219:183–93. doi: 10.1002/jcp.21669, PMID: 19097143

[58] Jarosz M Olbert M Wyszogrodzka G Mlyniec K Librowski T. Antioxidant and anti-inflammatory effects of zinc. Zinc-dependent NF-kappa B signaling. Inflammopharmacology. (2017) 25:11–24. doi: 10.1007/s10787-017-0309-4 28083748 PMC5306179

[59] Cavallini D Raspa F Marliani G Nannoni E Martelli G Sardi L. Growth performance and feed intake assessment of Italian Holstein calves fed a hay-based total mixed ration: preliminary steps towards a prediction model. Vet Sci (Basel). (2023) 10:554. doi: 10.3390/vetsci10090554, PMID: 37756076 PMC10536390

[60] Raspa F Tarantola M Muca E Bergero D Soglia D Cavallini D. Does feeding management make a difference to behavioural activities and welfare of horses reared for meat production? Animals (Basel). (2022) 12:1740. doi: 10.3390/ani12141740, PMID: 35883287 PMC9311627

