# Supplementary material for: Effects of alkaline mineral complex supplementation on production performance, serum variables, and liver transcriptome in calves
Source: Front Vet Sci. 2023 Dec 6;10:1282055. doi: 10.3389/fvets.2023.1282055 (PMC10730931; doi:10.3389/fvets.2023.1282055)
Supplement: Supplementary file 1 [file Data_Sheet_1.docx]

Supplementary Material

# Supplementary Figures and Tables

##
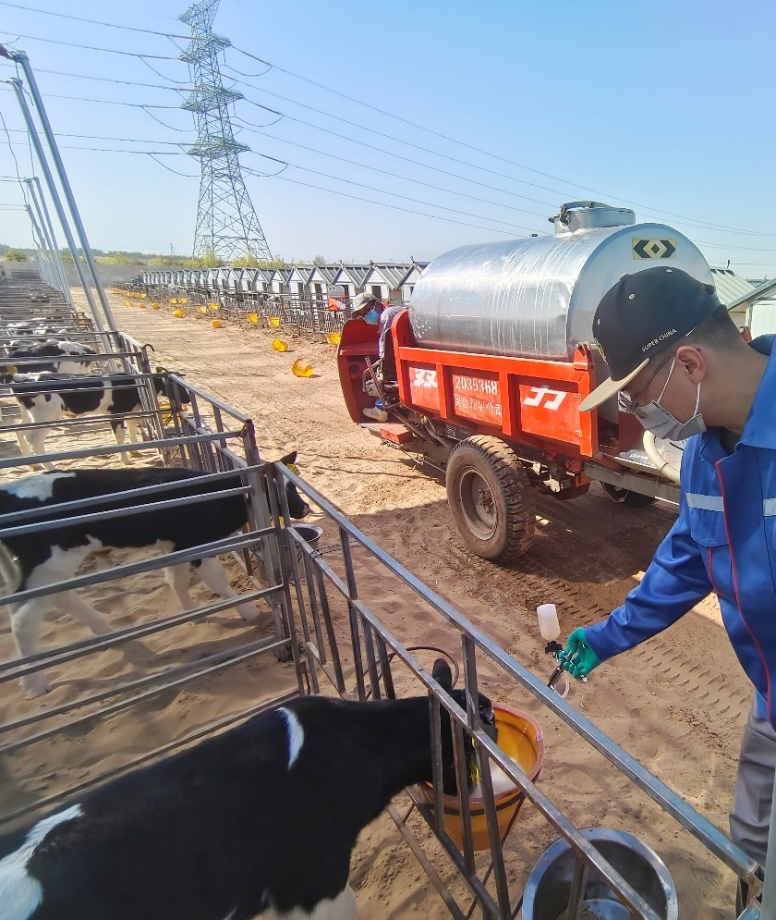
Supplementary Figures

**Figure S1.** Supplement method of alkaline mineral complex.

**1.2 Supplementary tables**

**Table S1**$.$Nutritional composition of granules.

| Nutrient component | Guarantee Value% |
| --- | --- |
| CP | ≥20.00 |
| CF | ≤12.00 |
| Ash | ≤9.00 |
| Ca | 0.70–1.80 |
| Total P | ≥0.40 |
| NaCl | 0.30–1.50 |
| Lysine acuity | ≥0.70 |
| Water | ≤14.00 |

CP is crude protein; CF is crude fiber; Ca is calcium; NaCl is sodium chloride.

Ingredients: corn, soybean meal, cotton meal, corn dry alcohol grains, corn germ meal, corn husk, vitamin D3, vitamin A acetate, copper sulfate, manganese sulfate, DL-α-tocopherol acetate.

**Table S2**$\mathbf{.}$Nutritional composition of milk replacer

| Nutrient component | Guarantee Value% |
| --- | --- |
| CP | ≥21.00 |
| EE | ≥16.00 |
| CF | ≤0.30 |
| Ash | ≤10.00 |
| Ca | ≥0.30 |
| Total P | ≥0.30 |
| NaCl | ≥1.00 |
| Water | ≤6.00 |
| Lysine acuity | ≥1.00 |
| Lactose | 38–46% |
| Vitamin A | ≥5000IU/Kg |

CP is crude protein; CF is crude fiber; EE is ether extract; Ca is calcium.

Ingredients: Whey protein powder, coconut oil, palm oil, wheat hydrolyzed protein, Vitamin A.

**Table S3**$.$Nutritional composition of normal milk and feeding milk

| Nutrient component | Normal milk % | Feeding milk % |
| --- | --- | --- |
| CP | 2.90 | 3.25 |
| EE | 3.50 | 3.40 |
| Lactose | 5.20 | 5.30 |
| Total Solid | 13.30 | 13.65 |

CP is crude protein; EE is ether extract.

The mixing ratio of feeding milk is: (milk replacer 1: water 7): normal milk =1:1.
